# Suppression of Inflammation Delays Hair Cell Regeneration and Functional Recovery Following Lateral Line Damage in Zebrafish Larvae

**DOI:** 10.3390/biom10101451

**Published:** 2020-10-16

**Authors:** Ru Zhang, Xiaopeng Liu, Yajuan Li, Ming Wang, Lin Chen, Bing Hu

**Affiliations:** 1 Hefei National Laboratory for Physical Sciences at the Microscale, CAS Key Laboratory of Brain Function and Disease, School of Life Sciences, Division of Biomedical Sciences, University of Science and Technology of China, Hefei 230027, China; zhangru@ustc.edu.cn (R.Z.); wming@ustc.edu.cn (M.W.); 2 Laboratory of Neurodevelopment and Repair, University of Science and Technology of China, Hefei 230027, China; lyj106@ustc.edu.cn; 3 Center for Hearing and Deafness, State University of New York at Buffalo, Buffalo, NY 14214, USA; xpliu8@mail.ustc.edu.cn; 4 Auditory Research Laboratory, University of Science and Technology of China, Hefei 230027, China

**Keywords:** hair cell regeneration, neuromast, zebrafish larva, rheotaxis, calcium imaging

## Abstract

Cochlear hair cells in human beings cannot regenerate after loss; however, those in fish and other lower species can. Recently, the role of inflammation in hair cell regeneration has been attracting the attention of scientists. In the present study, we investigated how suppression of inflammatory factors affects hair cell regeneration and the functional recovery of regenerated hair cells in zebrafish. We killed hair cells in the lateral line of zebrafish larvae with CuSO_4_ to induce an inflammatory response and coapplied BRS-28, an anti-inflammatory agent to suppress the inflammation. The recovery of the hair cell number and rheotaxis was slower when CuSO_4_ and BRS-28 were coapplied than when CuSO_4_ was applied alone. The recovery of hair cell count lagged behind that of the calcium imaging signal during the regeneration. The calcium imaging signal in the neuromasts in the inflammation-inhibited group was weaker than that in the noninflammation-inhibited group at the early stage of regeneration, although it returned to normal at the late stage. Our study demonstrates that suppressing inflammation by BRS-28 delays hair cell regeneration and functional recovery when hair cells are damaged. We suspect that BRS-28 inhibits pro-inflammatory factors and thereby reduces the migration of macrophages to delay the regeneration of hair cells.

## 1. Introduction

Deafness and hearing defects are usually caused by the loss of sensory hair cells. The loss of hair cells can result from aging, infection, genetic factors, hypoxia, autoimmune disorders, ototoxic drugs, or noise exposure. Unfortunately, hair cells cannot regenerate in mammals, including humans [1,2], which leads to permanent hearing loss or impairment. In contrast, hair cells in some nonmammalian vertebrates, such as birds, reptiles, amphibians, and fish, have a remarkable ability to regenerate [3,4,5,6,7]. When hair cells are damaged, supporting cells transdifferentiate into both hair cells and supporting cells, or convert into hair cells directly in the species with the capacity of hair cell regeneration [8,9,10,11]. Scientists have a strong interest in decoding the mechanism underlying hair cell regeneration in these species in the hope that they will someday achieve hair cell regeneration in mammals.

Extensive studies have been carried out in an attempt to decode the mechanisms governing hair cell regeneration, which is now known to be regulated by the interaction of multiple signaling pathways, such as Notch signaling, Wnt/b-catenin signaling, Fgf signaling, and retinoic acid [12,13,14,15,16,17,18]. While a great deal of attention has been paid to varied signal pathways, the role of inflammation in hair cell regeneration has been largely overlooked. In fact, the process of hair cell damage is accompanied by an extensive inflammatory reaction. Inflammation has been found to play a role in tissue regeneration in recent years [19]. For example, macrophages are considered to have a key function in the inflammatory resolution stage, and are needed for fin regeneration and hair cell regeneration in zebrafish [20,21]. In addition, it has been confirmed that neutrophils in mice play a central role in inflammation-induced optic nerve regeneration [22]. However, relatively little study on the role of inflammation in hair cell regeneration is available in the literature.

In recent years, the zebrafish (*Danio rerio*) has become an ideal model for studying inflammation and hair cell regeneration because it has conservative innate immunity and strong regeneration ability in the lateral line system [23,24], a sensory organ which perceives changes in the surrounding flow in order to detect prey and avoid predators [25,26]. The lateral line system of a larva is composed of neuromasts, which are located on the surface of the body. The neuromasts on the head constitute the anterior lateral line (aLL) system, and the ones along the body comprise the posterior lateral line (pLL) system [27]. The center of the neuromast is composed of hair cells, which are surrounded by supporting cells and mantle cells. At the top of the hair cells, rows of short stereocilia and a long kinocilium extend out of the body and are covered in a gelatinous cupula, which is called the hair bundle. The arrangement of stereocilia and kinocilia determines the polarity of hair cells, and the polarity of the hair cells is planar cell polarity, which is arranged symmetrically [28], i.e., half in each direction.

When hair bundles are deflected, hair cells release neurotransmitters and cause exciting spikes in afferent neurons [26]. Then, larvae show a robust sprint called rheotaxis [29]. This behavior can be used to evaluate the function of hair cells [30]. However, at present, no ideal behavioral methods are available for evaluating the function of lateral hair cells. Usually, zebrafish larvae are placed in accelerating flow to observe whether they have positive rheotaxis behavior [29,30,31]. However, this can only assess whether larvae have rheotaxis, and the stimulation of accelerating flow is relatively high, which is not conducive to the accurate assessment of the function of hair cells. A new behavioral device should be developed for evaluating rheotaxis quantitatively.

In recent years, calcium imaging has become a popular method to measure the function of neural cells quantitatively and in detail [32]. It enables measurements of mechanically evoked activity in all of the individual hair cells in a neuromast to be made simultaneously. When the mechanical hair bundle deflects, calcium and other cations enter the cytoplasm through mechanotransduction channels. This changes the membrane potential and activates voltage-gated calcium channels, which allow rapid calcium inflow to trigger synaptic transmission. GCaMPs, a type of genetically encoded calcium indicator (GECI), are single fluorescent proteins that can bind calcium directly and alter conformation to respond to changes in calcium concentration [33]. These significant, activity-dependent signals can reflect the function of hair cells in a single neuromast [32,34].

Few studies are available on the role of inflammation in hair cell regeneration. While a recent study reported that the deletion of macrophages by morpholinos led to the delay of hair cell regeneration [21], we were interested in whether or not suppression of pro-inflammatory factors affected hair cell regeneration and, if so, whether it affected the functional recovery of regenerated hair cells. In order to answer these questions, we used BRS-28, an anti-inflammatory agent, to suppress the production of pro-inflammatory factors when hair cells were damaged by copper. Unlike some classic anti-inflammatory drugs, such as cortisol or aspirin, that can affect the development of zebrafish larvae [35,36], BRS-28 shows weak or no cell toxicity, but exhibits potent inhibitory activities on nitric oxide production in LPS-stimulated BV-2 microglia cells [37]. In our study, we counted the number of neutrophils and macrophages in the *Tg*(*coro1a:eGFP; lyz:Dsred2*) transgenic line. Then, we used the wild-type AB strain zebrafish larvae to count the number of regenerated hair cells. Since there is no appropriate behavioral method to quantitatively evaluate the function of lateral line hair cells, we developed a device to test rheotaxis behavior in wild-type AB larvae. We also developed behavioral analysis software for quantitative evaluation of rheotaxis so as to reflect the holistic functional recovery of the posterior lateral line. Finally, we evaluated the function of the regenerated hair cells in a single neuromast with calcium imaging in the *Huc:h2b-gcamp6f* transgenic line.

## 2. Materials and Methods

### 2.1. Zebrafish Strains and Maintenance

A wild-type AB strain and *Tg*(*coro1a:eGFP; lyz:Dsred2*) and *Huc:h2b-gcamp6f* transgenic lines were used in this study. *Tg*(*coro1a:eGFP; lyz:Dsred2*) double transgenic line was generated by mating F1 *Tg*(*coro1a:eGFP*) with *Tg*(*lyz:Dsred2*) fish. *Huc:h2b-gcamp6f* was expressed as pan-neuronal nucleus-labeled GCaMP6f. Embryos were generated by paired mating and maintained at 28.5 °C in 10% Hanks’ solution (137 mM NaCl, 5.4 mM KCl, 1 mM MgSO_4_, 0.44 mM KH_2_PO_4_, 0.25 mM Na_2_HPO_4_, 4.2 mM NaHCO_3_, 1.3 mM CaCl_2_ for 100% solution, adjusted to pH 7.3 with NaOH) under a 14/10 h light/dark cycle, according to the standard protocols [38]. All animal manipulations were conducted strictly in accordance with the guidelines and regulations set forth by the University of Science and Technology of China (USTC) Animal Resources Center and the University Animal Care and Use Committee. The protocol was approved by the Committee on the Ethics of Animal Experiments of the USTC (Permit Number: USTCACUC1103013).

### 2.2. Hair Cell Damage and TUNEL Assay

In order to damage hair cells in the lateral line, we treated the larvae four days postfertilization (dpf) with 5 μM CuSO_4_ (Sangon, Shanghai, China) diluted in 10% Hanks’ solution for 1 h. Then, we washed them three times and allowed them to recover in 10% Hanks’ solution.

TUNEL (TdT-mediated dUTP Nick-End Labeling) assay was used to confirm apoptosis of hair cells. After being treated with 5 μM CuSO_4_ for 0, 20,40 and 60 min respectively, larvae were fixed with 4% paraformaldehyde for 2 h at room temperature. Using the TUNEL kit (Vazyme, Nanjing, JS, China), according to the manufacturer’s instructions, we stored the fixed larvae overnight at 4 °C. The staining solution was removed with PBS. After finding the location of the neuromasts in the bright field channel, a superimposed image was taken under a confocal microscope (ZEISS 710, Zeiss, Oberkochen, RS, Germany) with different excitation wavelengths at the same optical section.

### 2.3. Inflammation Inhibition

To suppress the inflammation in a preliminary experiment, we assessed the anti-inflammatory effect of BRS-28 in the classic tail fin amputation experiment at different concentrations and different treatment times (data not shown). Based on the results, we determined that the optimal working concentration of BRS-28 was 20 μM and the optimal treatment time was 3 h before moving zebrafish larvae into CuSO_4_ to damage hair cells.

### 2.4. Live Imaging

**Figure 1:**
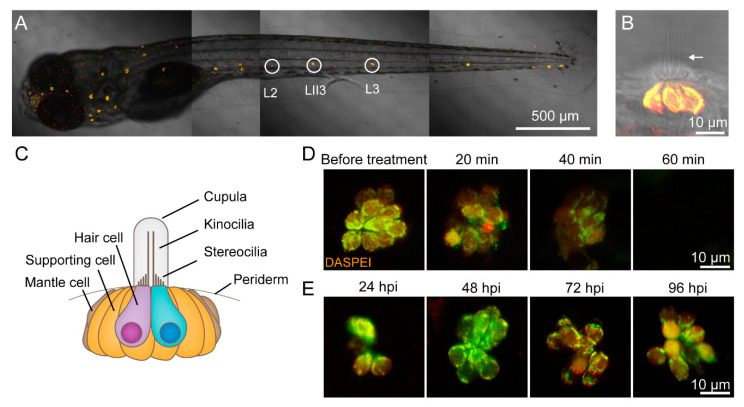
CuSO_4_ damages hair cells in the lateral line of zebrafish. (**A**) Lateral line hair cells in a 6 days postfertilization (dpf) wild-type AB zebrafish larva is labeled with 0.05% DASPEI. L2, LII3, and L3 neuromasts are marked with circles. Scale bar represents 500 μm. (**B**) The lateral view of a neuromast shows sensory hair cells in the center labeled with DASPEI and a bundle of kinocilia (arrow) extending out of the periderm. Scale bar represents 10 μm. (**C**) A cartoon illustrates the structure of the neuromast. (**D**) Time lapse imaging shows that when immersed in 5 μM CuSO_4_ solution, hair cells were gradually injured and damaged within 60 min. Scale bar represents 10 μm. (**E**) DASPEI staining displays that hair cells regenerate completely within 96 h postinjury (hpi). Scale bar represents 10 μm.

Wild-type AB larvae were used to count the number of regenerated hair cells in L2, LII3, and L3 neuromasts (Figure 1A). Hair cells were marked by 0.01% DAPI (Invitrogen, Carlsbad, CA, USA) for 5 min. Larvae were anesthetized in 0.02% MS-222 (Tricaine mesylate, Sigma-Aldrich, St. Louis, MO, USA) and imaged under a fluorescence microscope (BX-60, Olympus, Tokyo, Japan).

In order to exhibit the damage of hair cells in copper sulfate solution and the regeneration of hair cells in different phases, we labeled hair cells with 0.05% 2-[4-(Dimethylamino)styryl]-1-ethylpyridinium iodide (DASPEI) (Sigma-Aldrich, St. Louis, MO, USA). The larvae were anesthetized in MS-222 and imaged under a confocal microscope (LSM 880 +Airyscan, Zeiss, Oberkochen, RS, Germany).

The *Tg*(*coro1a:eGFP; lyz:Dsred2*) transgenic line was used to show the number of neutrophils and macrophages migrating to the injured neuromasts in vivo. In this transgenic line, neutrophils coexpress *lyz:Dsred2* and *coro1a:GFP* and show yellow fluorescence after these two channels are merged, while macrophages only express *coro1a:GFP* and show green fluorescence [20]. To show the neutrophils and macrophages migrating to damaged neuromasts, we anesthetized the larvae in MS-222 and imaged them using a confocal microscope (LSM 880 +Airyscan, Zeiss, Oberkochen, RS, Germany). In order to count the neutrophils and macrophages, we set the area around the L2, LII3, and L3 neuromasts with a diameter of 100 μm as the region of interest (ROI). The zebrafish larvae were anesthetized and imaged by a fluorescence microscope (BX-60, Olympus, Tokyo, Japan) with a green and a red channel. The data at each time point were from different zebrafish larvae. When a few L2, LII3, or L3 neuromasts were unrecognizable because of damage to the hair cells, the data from these neuromasts were excluded.

### 2.5. Rheotaxis Behavioral Experiments

A U-shaped tank was designed for testing the rheotaxis behavior of larvae. The bottom of the two cubic tanks (7 cm length × 8 cm width × 8 cm height) were connected by a platform (10 cm length × 8 cm width × 0.5 cm height). A peristaltic pump (Longer Pump YZ1515x, Longer, Baoding, HB, China) was used to move 10% Hanks’ solution from the left tank to the right tank, so that the platform formed a steady water flow from right to left. The velocity of water flow was 8 mm/s, which was enough to stimulate hair cells to produce signals [39]. Wild-type AB zebrafish larvae were used for detecting the rheotaxis ability. The larvae were released at the right side of the platform, and the relative velocity of water flow was close to zero. To avoid visual cues, we performed the experiments in dark, and recorded rheotaxis with an infrared CCD (IR850, Weixinshijie, Shenzhen, GD, China). The fish for behavioral experiments were allocated into four groups: the control group, BRS group, CuSO_4_ group and BRS+CuSO_4_ group. Each group had 50 larvae. After the hair cells in CuSO_4_ and BRS+CuSO_4_ groups were damaged, tests of rheotaxis were conducted on the larvae in these four groups, and the tests were repeated for the same larvae every 24 h thereafter. A very small number of data were eliminated when of larvae died or data were disturbed by bubbles.

Rheotaxis data were analyzed by our rheotaxis software developed in Matlab (2015a, MathWorks, Natick, MA, USA) (Videos S1 and S2). With this software, we could plot the movement track of zebrafish larvae in the platform, measure the direction and distance of each sprint, and calculate the speed. Finally, the software could report scores based on the ratio of the projection of the motion vector sum to the 0° direction to the arithmetic sum of the motion. If this ratio was negative, the score was treated as zero.

### 2.6. Calcium Imaging and Data Analyses

The *Huc: h2b-gcamp6f* transgenic line, which expressed pan-neuronal, nucleus-labeled GCaMP6f, was used in calcium imaging. The larvae were anesthetized and fixed by a net pressure. The one-step pulled micropipette had a long, wispy tip that had been trimmed by rubbing it against another pulled micropipette to generate a tip with an outer diameter of approximately 40 μm. The micropipette was filled with 0.02% MS-222 and fixed to the holder of a micromanipulator (MP225, Sutter, Novato, CA, USA). The tip of the micropipette was positioned at a distance of approximately 100 μm from the top of the kinocilia. The duration of flow was controlled by three direct links, which were linked with a syringe.

Calcium imaging was collected by a confocal microscope (FV 1000, Olympus, Tokyo, Japan). To include as many hair cells as possible in the observation area at the same time, we adjusted a single *z*-axis. The observed area was set to 110 × 108. We took 100 time-lapse images for each neuromast, and the total capture time was 29.7 s (0.297 s per slice). Flow stimulation occurred from 10.098 to 19.899 s.

The neuromasts are three-dimensional, and different hair cells have different levels of fluorescence intensity. Therefore, relative fluorescence intensity changes (F = ΔF/F_0_) are more commonly used [32]. We selected an ROI for each hair cell, and the average fluorescence intensity in the ROI obtained before applying flow stimuli (0–10 s) was set as the baseline fluorescence intensity (F_0_). The baseline fluorescence intensity was subtracted from each frame of the ROI to obtain the change in fluorescent signal from the baseline, which represents ΔF. The data were excluded when F_0_ < 95, which indicated that these hair cells were too far from the focal plane. When more than two hair cells in the neuromast responded to flow stimulation, the two hair cells with the strongest ΔF/F_0_ were selected and included in the statistics of the temporal curve of the fluorescence intensity.

### 2.7. Statistical Analysis

All data are shown as the mean ± S.E.M. or as relative proportions of 100% as indicated in the appropriate legends. The data were analyzed using either one-way ANOVA with Tukey’s multiple comparisons test or two-way ANOVA with Tukey’s multiple comparisons test in GraphPad Prism version 7.0 (Prism, San Diego, CA, USA). The level of significance was set to *p* < 0.05.

## 3. Results

### 3.1. Hair Cells Regenerated in the Lateral Line of Zebrafish Following CuSO_4_ Toxicity

Figure 1A shows a sample image of sensory hair cells in a 6 dpf wild-type AB zebrafish larva labeled with 0.05% DASPEI. Three of the posterior lateral neuromasts were L2, LII3, and L3 neuromasts (circles in Figure 1A) located along the flat trunk body and were clearly visible. A lateral view of the neuromasts at high magnification showed the elongated kinocilia extending from the body (Figure 1B). The neuromasts consist of hair cells surrounded by supporting cells, which are surrounded by mantle cells (Figure 1C).

In order to study the role of inflammation in hair cell regeneration, we developed a hair cell toxicity model in which hair cells were completely destroyed when treated with 5 μM CuSO_4_ for 1 h (Figure 1D). Before the treatment, hair cells displayed close arrangement and clear boundaries, as labeled with 0.05% DASPEI. When treated with CuSO_4_ for 20 min, hair cells became loose and blurred, indicating injury. When treated with CuSO_4_ for 40 min, the number of surviving hair cells decreased and cell boundaries were obscure, as indicated by weaker fluorescence intensity. When treated with CuSO_4_ for 60 min, hair cells completely disappeared. Under a confocal microscope, it could be seen that larvae of *Huc:h2b-gcamp6f* transgenic lines had green, fluorescently labelled nuclei of hair cells. The fluorescence disappeared after treated with 5 μM CuSO_4_ for 1 h, which indicated that hair cells were damaged (data not shown). A TUNEL assay suggested that the missing hair cells had undergone apoptosis (Supplementary Materials Figure S1).

We then transferred the zebrafish with destroyed hair cells in the lateral line to 10% Hanks’ solution and allowed them to recover. At 24 h postinjured (hpi), the newly regenerated hair cells were visible. At 48 and 72 hpi, more hair cells regenerated. At 96 hpi, hair cells had fully regenerated (Figure 1E).

### 3.2. Anti-Inflammatory Agent Reduced the Number of Neutrophils and Macrophages Migrating to the Injured Neuromasts

**Figure 2:**
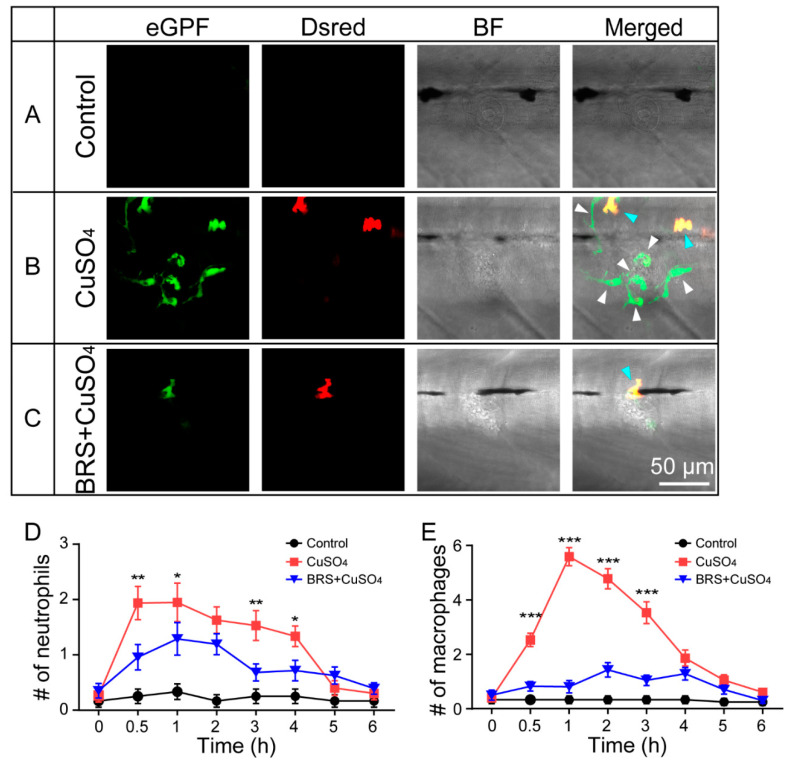
BRS-28 reduces the number of neutrophils and macrophages migrating to the injured neuromasts. (**A**–**C**) Live imaging (40×) displays the regions of L3 neuromasts of larvae at the GFP channel, Dsred channel, and bright field (BF) channel and a superimposed image in different groups. Neutrophils (showing both green and yellow fluorescence, indicated by blue arrows) and macrophages (showing only green fluorescence, indicated by white arrows) around the neuromasts could be observed in *Tg*(*coro1a:eGFP; lyz:Dsred2*) larvae. They were almost entirely absent from the neuromasts in the control group (**A**). Many neutrophils and macrophages migrated to injured neuromasts in the CuSO_4_ group (**B**), while fewer neutrophils and macrophages migrated to injured neuromasts in the BRS+CuSO_4_ group (**C**). The image was captured after adding CuSO_4_ solution for 1 h. Scale bar represents 50 μm. (**D**,**E**) Line charts reveal decreased numbers of neutrophils (**D**) and macrophages (**E**) within a radius of 50 μm from the center of neuromasts at different time points after adding CuSO_4_ in the BRS+CuSO_4_ group (16 ≤ *n* ≤ 23) compared to the CuSO_4_ group (15 ≤ *n* ≤ 23). The control group (11 ≤ *n* ≤ 12) was observed at the same time points. The *y*-axis is a mean of multiple neuromasts (L2, LII3, and L3 neuromasts) from multiple zebrafish. The *n* values represent the numbers of neuromasts. In (**D**,**E**), the asterisk shows the difference between the CuSO_4_ group and the BRS+CuSO_4_ group. Comparisons were performed using two-way ANOVA, with Tukey’s multiple comparisons test. All error bars show mean ± S.E.M., *** *p* < 0.001, ** *p* < 0.01, * *p* < 0.05.

Neutrophils (Figure 2B,C, blue arrows) and macrophages (Figure 2B,C, white arrows) could be marked and distinguished in the larvae of the *Tg*(*coro1a:eGFP; lyz:Dsred2*) transgenic line (Figure S2). In the control group, neutrophils and macrophages were hardly observed around the neuromasts (Figure 2A). In the CuSO_4_ group, in which hair cells were damaged, neutrophils and macrophages migrated to the neuromasts in 1 h (Figure 2B,D). In the BRS+CuSO_4_ group, the larvae were immersed in BRS-28, an anti-inflammatory agent, before treatment with CuSO_4_ solution. In this group, fewer neutrophils and macrophages migrated to the damaged neuromasts (Figure 2C,E). The number of neutrophils appearing around the damaged neuromasts 0.5, 1, 3, and 4 h posttreatment was significantly lower in the BRS+CuSO_4_ group than in the CuSO_4_ group (Figure 2D). Similarly, the number of macrophages appearing around the damaged neuromasts 0.5, 1, 2, and 3 h posttreatment was significantly lower in the BRS+CuSO_4_ group than the CuSO_4_ group (Figure 2E). These results indicate that BRS-28 reduces the number of neutrophils and macrophages migrating to the injured neuromasts. It is worth noting that 5 and 6 h posttreatment, neutrophils and macrophages in either the CuSO_4_ group or the BRS+CuSO_4_ group were not significantly different in number from those in the control group, indicating that the inflammation had been resolved.

### 3.3. Anti-Inflammatory Agent Delayed Hair Cell Regeneration

**Figure 3:**
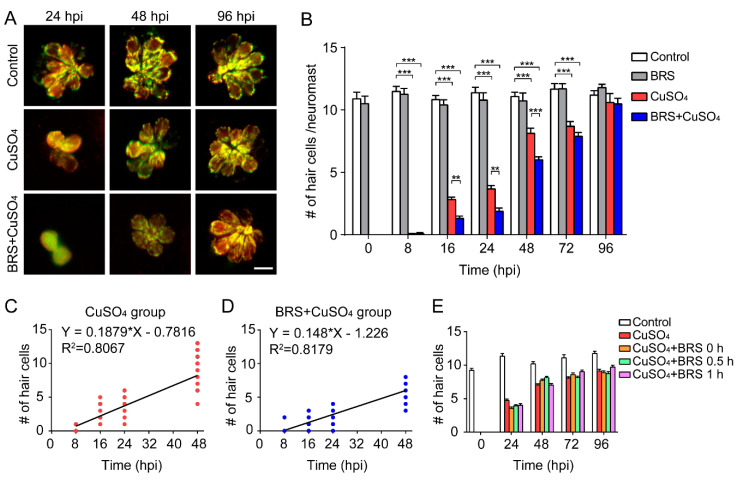
Suppressing inflammation delays hair cell regeneration. (**A**) Real-time imaging (40×) displays regenerated hair cells in the CuSO_4_ and BRS+CuSO_4_ group at 24, 48, and 96 hpi. The control group was imaged at the same time points. Scale bar represents 10 μm. (**B**) The numbers of regenerated hair cells were significantly lower in the BRS+CuSO_4_ group compared to the CuSO_4_ group at 16 (*n* ≥ 27, *p* < 0.01), 24 (*n* ≥ 21, *p* < 0.01), and 48 (*n* ≥ 24, *p* < 0.001) hpi. At 96 hpi, hair cells in both the CuSO_4_ group and the BRS+CuSO_4_ group had regenerated to normal levels. Linear analysis in the CuSO_4_ group (**C**) and BRS+CuSO_4_ group (**D**) was conducted on the number of regenerated cells within 48 hpi. The slope in the CuSO_4_ group (0.188) was higher than that in the BRS+CuSO_4_ group (0.148), and the x-intercept in the CuSO_4_ group (4.160) is higher than that in the BRS+CuSO_4_ group (8.287). (**E**) When the time window of inflammatory suppression was delayed, there was no delay in the regeneration of hair cells. BRS-28 was added at the same time as CuSO_4_ (CuSO_4_+BRS 0 h group), 30 min after the addition of CuSO_4_ (CuSO_4_+BRS 0.5 h group), or 1 h after the addition of CuSO_4_ (CuSO_4_+BRS 1 h group) (*n* ≥ 27 neuromasts at each time point of each group). For (**B**) and (**E**), comparisons were performed using two-way ANOVA, with Tukey’ multiple comparisons test. All error bars show the mean ± S.E.M., *** *p* < 0.001, ** *p* < 0.01.

In order to investigate what happens to hair cell regeneration after inflammation is suppressed, we observed hair cells in the L2, LII3, and L3 neuromasts with live imaging in the control group, in the BRS group, in the CuSO_4_ group, and in the BRS+CuSO_4_ group at different phases. Sample imaging of hair cells in the control group, in the CuSO_4_ group, and in the BRS+CuSO_4_ group at 24, 48, and 96 hpi is shown in Figure 3A. There were significantly fewer regenerated hair cells in the BRS+CuSO_4_ group than in the CuSO_4_ group at 16 hpi (*p* < 0.01), 24 hpi (*p* < 0.01), and 48 hpi (*p* < 0.001) (Figure 3B, *n* ≥ 21 neuromasts). However, there was no significant difference in the number of hair cells at 96 hpi between the control group and the CuSO_4_ group, and between the control group and the BRS+CuSO_4_ group. Hair cells in the BRS group were not significantly different from those in the control group at any phase (Figure 3B). These results indicate that the regeneration of hair cells was delayed after the inflammation had been suppressed by the anti-inflammatory agent BRS-28.

Since hair cells did not regenerate at a uniform rate, we defined the time of regeneration into two periods: the Early Stage, which included the period from 0 to 48 hpi, and the Late Stage, which included the period after 48 hpi. The regeneration of hair cells was fast in the Early Stage and slow in the Late Stage. Linear analysis was conducted on the number of hair cells regenerated in the Early Stage. The slope in the CuSO_4_ group (0.1879) was higher than that in the BRS+CuSO_4_ group (0.148), while the x-intercept in the CuSO_4_ group (4.16) was higher than that in the BRS+CuSO_4_ group (8.287) (Figure 3C,D). These results imply that hair cell regeneration in the BRS+CuSO_4_ group began later and was slower than that in the CuSO_4_ group.

To explore whether the onset time of the inflammatory suppression is important to the delayed hair cell regeneration, we varied the onset time of BRS-28 treatment. BRS-28 was added at the same time as CuSO_4_ (CuSO_4_+BRS 0 h group), 0.5 h after the addition of CuSO_4_ (CuSO_4_+BRS 0.5 h group) and 1 h after the addition of CuSO_4_ (CuSO_4_+BRS 1 h group). There was no significant difference in the number of regenerated hair cells between the CuSO_4_ group and the three CuSO_4_+BRS groups (Figure 3E), indicating that the pace of hair cell regeneration would not be affected if the anti-inflammatory agent were applied after inflammation occurs.

### 3.4. Anti-Inflammatory Agent Delayed Recovery of the Impaired Rheotaxis Following CuSO_4_ Toxicity

**Figure 4:**
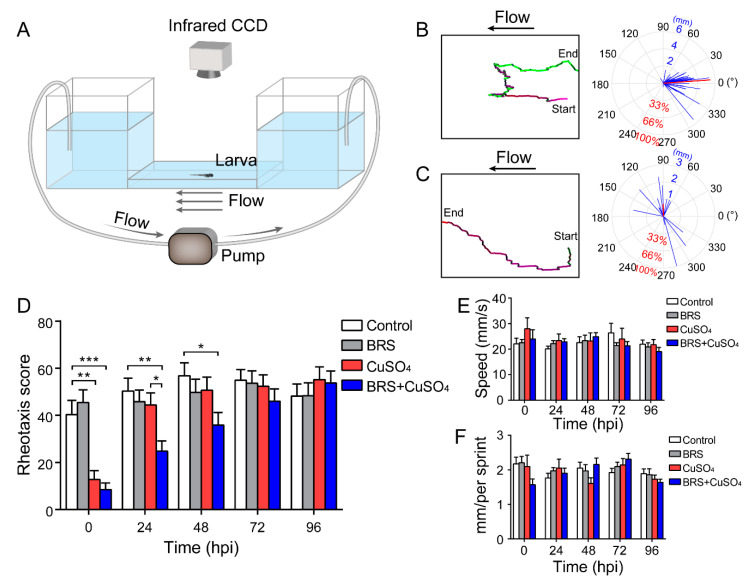
Assessment of rheotaxis reflects the function of the lateral line system. (**A**) A U-shaped tank was designed to test the rheotaxis behavior of larvae. A peristaltic pump was used to form the flow at the bottom of the tank. Larvae were placed in the right platform one by one, and they sensed the water flow from right to left. Rheotaxis was recorded by an infrared CCD. A larva with excellent rheotaxis (**B**) and a larva with poor rheotaxis (**C**) were analyzed by behavioral analysis software. Movement traces are plotted in the left panels, and the motion vectors are displayed in the right panels. The lengths of the blue segments represent the distance of each sprint, and the direction of the blue segment represents the direction of that sprint. The length of the red line segment is the ratio of the motion vectors’ sum to the motion arithmetic sum, and the direction is the direction of the sum of the vectors. (**D**) The rheotaxis score shows that at 0 hpi, both the CuSO_4_ and BRS+CuSO_4_ groups had poor rheotaxis. At 24 hpi, the rheotaxis of the BRS+CuSO_4_ group was significantly lower than that of the control group and CuSO_4_ group. Until 48 hpi, the rheotaxis score of the BRS+CuSO_4_ group was still significantly lower than that of the control group. On the contrary, the rheotaxis of the CuSO_4_ group was not significantly different from that of the control group within 24 hpi. *n* = 49, control group; *n* = 49, BRS group; *n* = 46, CuSO_4_ group; *n* = 47, BRS+CuSO_4_ group. The speed (**E**) and distance (**F**) of sprints were consistent across different times and between different groups. For (**D**–**F**), comparisons were performed using two-way ANOVA, with Tukey’s multiple comparisons test. All error bars show the mean ± S.E.M., *** *p* < 0.001, ** *p* < 0.01, * *p* < 0.05.

Since rheotaxis is a suitable functional readout of the lateral line, we developed a behavioral device to test the rheotaxis of zebrafish (Figure 4A; see Materials and Methods for details) in order to study the functional recovery of zebrafish lateral line following CuSO_4_ toxicity. In this device, larvae are placed in the right platform, and they sense the water flow direction from right to left. Here, we show two examples of the larval rheotaxis processed by our custom-developed behavioral analysis software: one is a larva with excellent rheotaxis (Figure 4B and Video S1), while the other is a larva with poor rheotaxis (Figure 4C and Video S2). The left panels in Figure 4B,C show the swimming track of the larvae. Our behavioral analysis software mapped the movement path of larvae by line segment. The color of the line segment represents the direction of movement. All the movements from right to left are represented by purplish or red segments, with purple indicating that the velocity along the flow direction is greater than or equal to the flow velocity, and with red indicating that the velocity along the flow direction is lower than the flow velocity. All the movements from left to right are represented by green segments, and the higher the brightness, the faster the speed. The right panels in Figure 4B,C display the motion vector. The lengths of the blue segments represent the distance of each sprint, and the direction of the blue segment represents the direction of that sprint. The length of the red line segment represents the ratio of the motion vector sum to the motion arithmetic sum, and the direction is the direction of the sum of the vectors.

Our behavioral analysis software reported a score as a measure of rheotaxis responses. A high score means good rheotaxis and a low score means poor rheotaxis. In the example (Video S1) in which the larva had good rheotaxis, as indicated by a long red segment with a small angle relative to the direction of flow (indicated by 0°), the motion of the larva was opposing the direction of water flow, indicating that its lateral line system executed its function very well. The software therefore reported a high score for this larva, i.e., 87.83 points. In the other example (Video S2) in which the larva had poor rheotaxis, as indicated by a short red segment with at a large angle relative to the direction of flow (0°), the motion of the larva was random, indicating a poor function of its lateral line system. In this case, the software reported a low score, i.e., −0.69 points.

With our device and analysis software, we examined the rheotaxis when the zebrafish larvae were treated with CuSO_4_ and/or BRS at 0, 24, 48, 72 and 96 hpi (Figure 4D) The rheotaxis scores in the CuSO_4_ group and in the BRS+CuSO_4_ group were significantly lower than those in the control group and in the BRS group at 0 hpi, indicating that these larvae had lost the ability to sense water flow. The rheotaxis score in the CuSO_4_ group was not significantly different from that in the control group and that in the BRS group at 24 hpi and thereafter. However, the rheotaxis score in the BRS+CuSO_4_ group was still significantly lower from that in the control group and that in the BRS group until 72 hpi. These results indicate that the anti-inflammatory agent BRS-28 delays functional recovery of the impaired lateral line system.

In order to exclude the possibility that BRS-28 and CuSO_4_ affect the function of the movement system, we examined the speed and the distance of each sprint of zebrafish in the control group, in the BRS group, in the CuSO_4_ group, and in the BRS+CuSO_4_ group. The was no significant difference in the speed (Figure 4E) or the distance (Figure 4F) of each sprint across the four groups and over the different times, indicating that BRS-28 or CuSO_4_ did not affect the sprint of zebrafish. In addition, the rheotaxis in the BRS group at each time point was not different from that in the control group, indicating that BRS-28 had no significant effect on the rheotaxis.

### 3.5. Calcium Imaging Revealed Recovery of Function of a Single Neuromast with Regenerated Hair Cells

**Figure 5:**
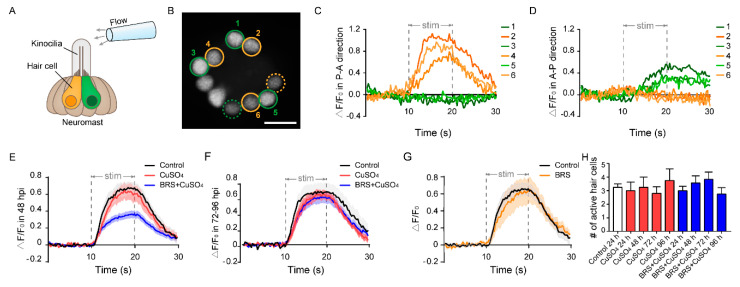
Calcium imaging reveals the function of a single neuromast. (**A**) The schematic diagram shows a glass micropipette filled with fluid located about 100 μm away from the top of kinocilia to stimulate the neuromast. The orange and green hair cells represent different polarities. (**B**) When stimulated by the flow, only a portion of hair cells responded in this focal plane (circled cells), and some were far from this focal plane (dashed circled cells). The No. 2, 4, and 6 active hair cells (orange circles) only responded to the flow in the P–A direction (**C**). At the same time, the No. 1, 3, and 5 active hair cells (green circles) only responded to the flow in the A–P direction (**D**). Scale bar in (**B**) represents 10 μm. (**E**) The relative fluorescence intensity change (ΔF/F_0_) of the BRS+CuSO_4_ group was significantly lower than that of the CuSO_4_ group in the Early Stage of regeneration (within 48 hpi) (*p* < 0.001). (**F**) ΔF/F_0_ of the BRS+CuSO_4_ group was not significantly different from that of the control group or CuSO_4_ group in the Late Stage of regeneration (72–96 hpi). (**G**) There was no difference in ΔF/F_0_ between the BRS group and the control group. (**H**) During the regeneration process, the numbers of active hair cells per neuromast in the CuSO_4_ and BRS+CuSO_4_ groups were basically the same and did not increase with the total number of regenerated hair cells. For (**E**–**H**), comparisons were performed using one-way ANOVA, with Tukey’s multiple comparisons test.

We found that recovery of impaired rheotaxis occurred much sooner than that of the number of regenerated hair cells. After the zebrafish lateral line was damaged by CuSO_4_, it took 96 h for the hair cells to return to normal (Figure 3B), while it took only 24 h for the rheotaxis to return to normal at 24 hpi (Figure 4D). To resolve this issue, we examined the function of a single neuromast with calcium imaging [32]. To acquire calcium images of hair cell activity, we stimulated the L3 neuromast, located in the flat trunk, with water flow from a glass micropipette (Figure 5A). Since hair cells of the L3 neuromast have polarities parallel to the anterior–posterior body axis [40], we controlled water flow in two directions: anterior to posterior (A–P) or posterior to anterior (P–A). The hair cells in orange circles and those in green circles in Figure 5B represent opposite polarities. We found that not all hair cells responded to the water flow, and only some were active (Figure 5B, circled cells). These active cells only responded to a stimulus in one direction, either the P–A direction (cells 2, 4, and 6 in Figure 5C, and cells in orange circles in Figure 5B) or the A–P direction (cells 1, 3, and 5 in Figure 5D, and cells in green circles in Figure 5B). Because the neuromasts were stereoscopic, some of the active hair cells were far from this focal plane (dashed circles in Figure 5B) and were not included in subsequent fluorescence intensity analyses.

Similar to the results of the rheotaxis, those of calcium imaging showed that ΔF/F_0_ in the regenerated hair cells was reduced significantly when inflammation was suppressed at the Early Stage of regeneration (within 48 hpi) (Figure 5E). In addition, ΔF/F_0_ in the CuSO_4_ group did not decrease significantly in the Early Stage. This might partially explain why the impaired rheotaxis in the CuSO_4_ group had recovered at 24 hpi. ΔF/F_0_ in the BRS+CuSO_4_ group was not significantly different from that in the control group and in the CuSO_4_ group in the Late Stage of regeneration (72–96 hpi) (Figure 5F). Additionally, ΔF/F_0_ showed no difference between the BRS group and the control group (Figure 5G), indicating that BRS-28 had no effect on calcium response.

Since only some of the hair cells in the neuromast responded to the stimulation of water flow [34], we wondered how many regenerated hair cells were active in the neuromast. We found that only a few regenerated hair cells in the CuSO_4_ group and in the BRS+CuSO_4_ group responded to flow stimuli. The numbers of active cells in each neuromast in these two groups were approximately the same at 24 to 96 hpi, and were consistent with those in the control group (Figure 5H).

## 4. Discussion

### 4.1. BRS-28 Suppressed Inflammation and Delayed the Initiation of Hair Cell Regeneration

Our study demonstrates that anti-inflammatory agent BRS-28 delays hair cell regeneration (Figure 3B) and delays functional recovery of the lateral line (Figure 4D and Figure 5E). BRS-28 dramatically reduced the number of neutrophils and macrophages migrating to the damaged neuromasts (Figure 2D,E) with no influence on larval movement (Figure 4E,F) or survival during the experiment. We found that when anti-inflammatory agent BRS-28 was applied after the application of CuSO_4_, there was no delay in hair cell regeneration (Figure 3E). This indicated that the timing of inflammation suppression is important. We think that when inflammation occurs, compensatory proliferation of the supporting cells is triggered and hair cells begin to regenerate. If inflammation suppression does not take effect, regeneration seems to be unaffected. This also implies that BRS-28 has a much greater effect on immune signals than other possible signals, such as regenerative signals.

What is the possible mechanism underlying the delay in recovery of hair cell regeneration when inflammation is suppressed? Kniss et al. proposed a hypothesis of triggering hair cell regeneration [41]. Studies of Drosophila wing discs and eyes have found that Jun N-terminal kinase (JNK) signaling pathways are required during apoptosis-induced compensatory proliferation [42,43,44]. Kniss et al. assumed that a similar process may be involved in the regeneration of hair cells. On the basis of this hypothesis, we speculate that when hair cells are damaged by CuSO_4_, it causes apoptosis in lateral line hair cells, triggers the rise of reactive oxygen species (ROS) and reactive nitrogen species (RNS), and induces oxidative stress. This process may improve activator protein-1 (AP-1), hypoxia-inducible factor 1-alpha (HIF-1α), and nuclear factor-κB (NF-κB) activity, and thus, increase pro-inflammatory cytokines and chemokines, such as NO, interleukin-1β (IL-1β), tumor necrosis factor (TNF)-α, and cyclooxygenase-2 (cox-2), inducible nitric oxide synthase (iNOS) [45]. BRS-28 is a derivative of 5α-cholestan-6-one, which has been confirmed to be a remarkable suppressor of the production of pro-inflammatory factors in a dose-dependent manner. It is reported that these compounds can significantly inhibit LPS-induced JNK and p38 phosphorylation and suppress the production of NO, TNF-α, IL-1β, cox-2, and iNOS [37,46,47]. Additionally, BRS-28 inhibits the production of pro-inflammatory factors, thereby further reducing macrophage activation. These processes decrease the production of TNF ligands and inhibit the JNK signal, which contribute to initiating cell regeneration and eventually lead to delayed initiation of compensatory proliferation and delayed regeneration of hair cells.

In addition, neutrophils can also remove dead cell debris, and macrophages can phagocyte apoptotic neutrophils or fragments of dead cells. We believe that when the number and activity of neutrophils and macrophages decrease, the clearance of damaged tissue areas slows down, and hair cells cannot obtain a good regeneration environment. Since damaged neuromasts need more time to clean up these cell fragments, this may also delay the regeneration of hair cells.

### 4.2. Suppression of Inflammation Delayed Functional Recovery of Regenerated Hair Cells

In this study, we found that when inflammation was suppressed, hair cell regeneration was delayed, as was the recovery of function. Finally, the quantity and the function of hair cells returned to a normal level at the Late Stage of regeneration. Therefore, although the suppression of inflammation delayed the regeneration of hair cells, it did not affect the overall process of hair cell regeneration, and the function of regenerated hair cells eventually tended to be intact. However, the effect of inflammation on the regeneration of lateral hair cells seemed to be different from that on the fin. Li et al. found that when zebrafish larvae lacked macrophages, vacuoles appeared in the regenerated fin, suggesting that macrophages may also be involved in fin regeneration [20]. In our research, although the suppression of inflammation delayed the regeneration of hair cells and their functional recovery at the Early Stage of regeneration, they eventually returned to the normal status at the Late Stage. This was not because inflammation was not suppressed sufficiently. Carrillo et al. used mutant strains, genetic ablation, or local ablation to obtain zebrafish larvae lacking neutrophils and/or macrophages, and found that the regeneration of the damaged hair cells was also delayed, but in the end, it was completed [21]. Moreover, the degree of delay in hair cell regeneration in their study was consistent with the results we obtained using anti-inflammatory agent BRS-28. The main function of hair cells of zebrafish is to sense the flow of water, and this function mainly depends on the hair cells in the pLL [29,39]. Therefore, in our research, we focused on the regeneration of hair cells in the pLL and evaluated its relationship with rheotaxis and calcium release under flow stimulation. Lateral line hair cells and tail fins have different regenerative capacities under inflammation inhibition, which may be because the injured organs are different, and the intact function of lateral hair cells is crucial for the survival of zebrafish. The hair cells in the lateral line may have more complex regulation mechanisms during the regeneration process.

### 4.3. Rheotaxis Was Restored before Hair Cells Had Fully Regenerated

Previous studies have focused on the morphological and quantitative recovery of regenerated hair cells in zebrafish [21,48]. Since regeneration takes 3–4 days postinjury, it is easy to assume that the recovery of the function of the neuromasts may be proportional to the number of regenerated hair cells. In this study, the CuSO_4_ group already showed excellent rheotaxis at 24 hpi (Figure 4D), even though the average number of hair cells was only 3.667 at that time (Figure 3B). Therefore, hair cells recover their function much faster than their numbers. In other words, although it takes 96 h to complete regeneration, the function of hair cells can recover within 24 h.

It is worth noting that the recovery of rheotaxis indicated that the kinocilia of the regenerated hair cells could sense small changes in water flow, and that the signal transmission pathway between the hair cells and the afferent nerves had formed. When BRS-28 was used to suppress the inflammation, the amplitude of the calcium activity of hair cells was significantly lower than that of the control and CuSO_4_ groups at the Early Stage of regeneration, and the rheotaxis of larvae was poor during this period. Therefore, the suppression of inflammation not only delayed hair cell regeneration but also delayed functional recovery.

### 4.4. The Number of Active Hair Cells Remained Relatively Constant as the Total Number of Hair Cells Increased during Regeneration

There was a discrepancy between normal function and the number of regenerated hair cells (Figure 3B and Figure 4D). Cell staining showed that the number of regenerative hair cells increased over time, and that regeneration was complete at 96 hpi (Figure 3B). However, the rheotaxis ability of the CuSO_4_ group returned to normal at 24 hpi, and at this time, there were only 3–4 regenerated hair cells per neuromast. Using calcium imaging and simultaneously measuring the mechanically induced activity of all hair cells in one neuromast, we found that only a small fraction of the hair cells responded to flow stimulation. Moreover, as the number of hair cells increased, the number of these active hair cells did not increase. Consistent with our findings on regenerated hair cells, Zhang et al. found that only some cells were active and responsive to stimulation, while other hair cells were silenced when they used calcium imaging to observe normal neuromasts [34]. This may be because gap junction-coupled supporting cells increase the intracellular K+ of silent cells, so that the Ca_v_1.3 channels of silent cells do not depolarize under stimulation. These silent cells may exist as backups. In our research, we found that this phenomenon also occurred in the regeneration group (CuSO_4_ and BRS+CuSO_4_ group). Regardless of the number of regenerated hair cells, the number of hair cells that responded to water flow remained stable during the regeneration process, which did not differ from the control group (Figure 5H). Additionally, in the Early Stage of regeneration, the magnitude of ΔF/F_0_ and reaction time of the CuSO_4_ group were also consistent with those of the control group. This explains why the number of regenerated hair cells in the CuSO_4_ group at 24 hpi was only 3.667 on average, but the function of the lateral line had been restored to a level very close to that of the control group.

In this study, we only performed calcium imaging on the L3 neuromast, which was confirmed as the polarity of the A–P body axis in the study of Chou et al. [40]. Consistent with their results, we found that this neuromast was indeed insensitive to the flow in the dorsal-ventral (D-V) body axis (data not shown). Therefore, this study only focused on the stimulus response in the A–P body axis direction and did not further analyze the stimulus data in the D-V body axis direction. Compared with hair cells with polarity in the A–P direction, hair cells with polarity in the P–A direction had greater ΔF/F_0_ when stimulated by water flow (Figure S3B; sample, Figure 5C,D). This indicated that the L3 neuromast is more sensitive to the flow from the P–A direction. This finding is consistent with the results obtained by Chou et al. using microphonic potentials evoked by sinusoidal stimuli [40]. Most active hair cells that respond to the opposite flow come in pairs (Figure S3A), suggesting that this behavior is pre-arranged rather than random.

## 5. Conclusions

Our study demonstrates that suppressing inflammatory factors by BRS-28 delays hair cell regeneration and functional recovery when hair cells are damaged, confirming that inflammation plays positive and permissive roles in hair cell regeneration. We suspect that BRS-28 inhibits pro-inflammatory factors and thereby reduces the migration of macrophages to delay the regeneration of hair cells. This study may provide new insights into how to promote hair cell regeneration and functional recovery in adult mammals.

## Supplementary Materials

The following are available online at https://www.mdpi.com/2218-273X/10/10/1451/s1, Figure S1: TUNEL assay detects apoptosis in hair cells, Figure S2: *Tg*(*coro1a:eGFP; lyz:Dsred2*) transgenic line can mark both neutrophils and macrophages, Figure S3: Most active hair cells are polar in pairs and are sensitive to flow in the P–A direction, Video S1: A larva with excellent rheotaxis is analyzed by the behavior analysis software, Video S2: A larva with poor rheotaxis is analyzed by the behavior analysis software.

## Abbreviations

aLL	anterior lateral line
AP-1	activator protein-1
A–P	anterior to posterior
BF	bright field
cox-2	cyclooxygenase-2
DASPEI	2-[4-(Dimethylamino)styryl]-1-ethylpyridinium iodide
dpf	day(s) postfertilization
D-V	dorsal-ventral
GECI	genetically encoded calcium indicator
HIF-1α	hypoxia-inducible factor 1-alpha
hpi	hour(s) postinjured
IL-1β	interleukin-1β
iNOS	inducible nitric oxide synthase
JNK	Jun N-terminal kinase
NF-κB	nuclear factor-κB
pLL	the posterior lateral line
P–A	posterior to anterior
RNS	reactive nitrogen species
ROI	region of interest
ROS	reactive oxygen species
TNF	tumor necrosis factor
TUNEL	TdT-mediated dUTP Nick-End Labeling
ΔF/F_0_	relative fluorescence intensity change
